# 

**Published:** 1920-12

**Authors:** 


### ANNOUNCEMENTS

### The Boston Session of the National Dental Association

The Twenty-fourth Annual Session of the National Dental Association was held in Boston, August 23-27, 1920. From the report of this session as given in the *Journal of the National Dental Association*, the meetings were a great success. The editorial in the above journal states that the session was a great meeting and is now a matter of history. We agree with the latter statement, but beg to amend the interpretation of the first.

From the standpoint of numbers present, membership, etc., the meeting might truthfully be said to have been the "biggest and best." The amount of money collected for various purposes and the advancement toward a permanent fund for the support of the *Journal of the National Dental Association*, all point to the interest manifested by the profession as a whole in the National Dental Association. Activities in the House of Delegates would tend to prove that the association existed for a purpose, even though the purpose might be the settling of whether "whiskey and brandy should be in the United States Pharmacopeia."

From the standpoint of the ordinary member, we do not believe that the success of a meeting can be judged by the activities in the House of Delegates or the amount of money collected by the secretary. Neither can success from a scientific standpoint be judged by these things. After the average man admits everything that the secretary calls attention to, and when he considers some of the things that were more evident in the meeting than the workings of the House of Delegates, he will be forced to the conclusion that the meeting was radically different from "the biggest and best," and will say, as we have heard many say, "This meeting is the worst I have ever attended." Yes, the Boston meeting, from the standpoint of the average member, was the worst we have ever seen, and we have seen several. Some of us who attended the New Orleans meeting thought things were about as bad as they could be. We heard several say at that meeting that they would never attend another, and, while conditions at New Orleans and Boston were not exactly alike, we prefer New Orleans.

Unless the meetings are planned differently in the future, they will not be representative of the dental profession. Very few men will attend year after year if they do not get more out of the meetings than they did at Boston. The sessions will be made up of men from the territory in which they are held.

From information gained from section officers of the association, we understand it is now difficult to get men to give papers. At New Orleans essayists complained that they could not get audiences. There seemed to be a tendency in the minds of some at New Orleans that the association was drifting into an organization that was more of a social affair than one given over to scientific research and the consideration of subjects of interest to dentistry. While the social activities were not made so prominent in Boston, it is safe to say that the scientific and practical sides of dentistry did not receive proper consideration. This was not exactly the fault of the officers of the association, but was because the meeting was planned without taking into consideration the fact that it was to be the "biggest and best" from the point of numbers; for it was the large attendance that ruined it, to a certain extent, as a scientific session.

A mistake was made in trying to hold all the sections in one building that was in no way suited for such a purpose. It would have been an ideal place for a political convention or Elks' Carnival, but as a meeting place for sections of a scientific organization, it was cut off by canvas and screens. The temporary partitions proberly equipped and separate rooms for each section as advertised by the advance press notices, the majority of the rooms were simply hallways that had been partitioned off by canvas and screens. The temporary partitons probably made a good appearance before the committee when six or eight people were in the building, but when a large number was present, so much noise and confusion could be heard through the various rooms that it was practically impossible to hear a paper read. It was also impossible to properly darken a room for the use of the stereopticon. The arrangements were so poor that one chairman refused to call his section to order amid the confusion, while other sections had to change rooms in order to hold their meetings at all. It sounds big to send out information that all sections of the National Dental Association will be held in one building; but the sad experience in Boston proved the fallacy of such a plan.

Another poor arrangement at the Boston meeting was the prominence given the dental dealers' and manufacturers' exhibit. In fact, it appeared that the Boston meeting was planned especially for the dental supply houses. We believe that the exhibits should be far enough away from the section meetings not to interfere with the scientific purposes of the organization. In Boston the entire lower floor of the building was given over to the commercial men while the upper floor was used for the association meetings; and every one who attended the scientific sessions or clinics was compelled to go through the section of exhibits. We have no grievance against dental supply men, and we believe if they are charged for space at the National Dental Association meetings, they should be given some time. However, as much as the dental supply man is a necessity, we believe he does not belong at the National Dental Association meetings or any other dental meeting. The National Dental Association is large enough not to have to be dependent upon exhibitors for money. If we are mistaken in this, we still contend that something must be done to keep the association from becoming a side attraction to the dental exhibit.

It has been previously mentioned that it is becoming difficult to get men to read papers before the National Dental Association, and this difficulty will increase unless more attention is given to the scientific sections. No one is going to try to listen to a paper amid such noise and confusion as existed at the Boston meeting. We hope that officers of the Association will profit by the mistakes of the Boston meeting, and not repeat the same thing at Milwaukee.—Editorial in October *International Journal of Orthodontia and Oral Surgery.*

---

### American Institute of Dental Teachers

The next annual meeting of the American Institute of Dental Teachers will be held at the Claypool Hotel, Indianapolis, Ind., January 24th, 25th and 26th, 1921. The program will contain much of interest in dental teaching methods and dental educational affairs. A

Chapman cordial invitation is extended to all ethical practitioners and others interested along these lines to attend the sessions. Dr. Arthur D. Black, President; Dr. Abram Hoffman, Secretary, 381 Linwood Ave., Buffalo, N. Y.

---

### Camp Roosevelt

The Board of Education of Chicago is endorsing a movement to do special work for the boys of this country which should interest our profession. You probably know that Camp Roosevelt was one of the big things in the middlewest during the summers of 1919 and 1920. It aroused more general interest than almost anything else in the country at that time. It is the greatest boys' summer camp in the world. The Board of Education of the City of Chicago and the U. S. War Department are behind the camp to the fullest extent, and it is planned to do the greatest amount of good for the greatest number of boys possible, by getting them out in the open, under most carefully safeguarded conditions.—F. L. Beals, Captain, U. S. Army.

